# Mapping of long COVID condition in India: a study protocol for systematic review and meta-analysis

**DOI:** 10.3389/fresc.2025.1419963

**Published:** 2025-02-13

**Authors:** Nidhi Jain, Komal Shah, Roshani Chauhan, Abhishek Gupta, Priyanka Arora, Deepak Saxena, Dileep Mavalankar

**Affiliations:** Department of Public Health Science, Indian Institute of Public Health, Gandhinagar, India

**Keywords:** COVID-19, long COVID, systematic review and meta-analysis, risk factors, health outcomes, chronic symptoms

## Abstract

**Background:**

The COVID-19 pandemic has reported significant alarming aftereffects experienced by some individuals following acute sequelae of SARS-CoV-2 infection, commonly referred to as long COVID. Long COVID is a set of symptoms that remain for weeks or months, after the initial phase of COVID-19 infection is ended.

**Objective:**

This study protocol outlines the methodology of a systematic review followed by a meta-analysis to comprehensively assess the chronic effects of COVID-19 infection on the Indian population and determine the likely risk factors connected to the development and persistence of long COVID.

**Methodology:**

This study will employ comprehensive search through a custom-made search strategy across significant databases (PubMed, MEDLINE etc.) and grey literature to identify related literature from January 2020 to December 2023. A systematic review and meta-analysis will be conducted to synthesize data from various studies. The data synthesis will involve a comprehensive narrative and tabular presentation of outcome data from included studies, focusing on long-term effects of COVID-19 infection in Indian population. A meta-analysis will be conducted contingent upon the availability and suitability of data. If sufficient and comparable quantitative data are identified across the included studies, statistical synthesis will be undertaken. Subgroup and sensitivity analyses will manage confounders, while MedCalc software will facilitate a meta-analysis to assess pooled data. Publication bias will be evaluated using statistical tests to ensure the integrity of the findings. In the absence of adequate data, a narrative synthesis will be performed to summarize the findings systematically and transparently.

**Conclusion:**

The anticipated findings will contribute to a refined understanding of this condition and its lingering symptoms, guiding healthcare interventions and future research endeavors to mitigate the impact of long COVID in the Indian population.

## 1 Introduction

Long COVID is identified as collective signs, symptoms, or problems that persist after acute COVID-19 infection. It is frequently called the post-COVID condition, long COVID, and post-COVID syndrome (1). Long COVID can include an extent of lingering problems that continue even after 3 months of SARS-CoV-2 infection, which might be new or returning, without any other explanation (2). However, long COVID is a diversified and clustered term that includes various symptoms that affect an individual's physical, cognitive, and psychological health. Also, the type and acuteness of symptoms vary from population to population. While some people recover gradually, others struggle with a long-lasting and frequently unpredictable course of symptoms that interfere with their day-to-day activity. Physical symptoms generally linked to long COVID include shortness of breath, persistent fatigue, joint pain, chest pain, headaches, heart palpitations, dizziness, gastrointestinal problems, or loss of taste or smell (3–6). Cognitive impairments such as memory problems, difficulty concentrating, mental fatigue, anxiety, depression, or mood swings collectively termed ‘brain fog’ can also be prevalent among those experiencing long COVID (7–9). Global estimates suggest that approximately 10%–20% of the population infected with COVID-19 has long-lasting symptoms of COVID-19 infection (10). Like other countries, India also encounters a significant proportion of the population suffering from aftereffects of COVID-19 infection, imposing a considerable burden on the healthcare system. With emerging evidence reporting an increasing burden of long COVID in Indians, it is imperative to (1) study, compile, and document the variety and severity of long COVID conditions/symptoms reported in Indians and (2) understand and estimate the actual burden of these conditions and identify the most shared symptoms prevalent in Indian population (3) study the effect of individual-level factors such as sociodemographic, clinical, and economic factors on the same. Here, the authors present a protocol for a systematic review followed by a meta-analysis (contingent upon the data) aiming to reveal the long-term effects of COVID-19 and assess the burden of long COVID in Indians.

## 2 Methods and plan of analysis

The study protocol presented here describes the methodology for a systematic review and meta-analysis to represent the long-term effects of COVID-19 on Indian population. The review was registered in the International Prospective Register of Systematic Reviews (PROSPERO) on 5 December 2023 (registration number CRD42023480037).

### 2.1 Eligibility criteria

#### 2.1.1 Inclusion criteria

The articles to be included are (1) original research articles, (2) published in English only, and (3) report empirical data [longitudinal or cohort studies (including short-term cohort studies) and cross-sectional studies are included] (4) published between January 2020 and December 2023 and (5) Studies involving participants of Indian ethnicity with a confirmed history of COVID-19 infection will be selected. Any inconsistencies will be addressed through a group discussion among the study team.

#### 2.1.2 Exclusion criteria

Individuals under 18 years of age, as well as pregnant and lactating women, will be excluded.

### 2.2 Search methods, information sources, study selection, and data management

A comprehensive electronic search will be developed using medical subject headings (MeSH terms), entry terms, and text words related to long COVID or post-acute sequelae of COVID-19. In addition, data search terms related to post effects, complications, symptoms, late stage, convalescent, sequelae, lingering effects, persistence, and consequences will also be used. The search strategy will be developed (customized to search database) and three databases will be explored, namely PubMed, Cochrane, and Ovid MEDLINE, from January 2020 to December 2023. In addition, the grey literature will also be searched. For grey literature, a thorough search across databases (such as Google Scholar and Grey Net) will be conducted to gather data. It will involve systematically identifying non-commercially published research or data to capture evidence on topic. Information from institutional repositories (like hospitals and government agencies), published reports, policy briefs from government agencies and NGOs, theses, preprints, conference proceedings, and documents from health organizations like WHO, ICMR, and Ministry of Ayush will be combined. Forward and backward citation searches will be included. Every database will have its own set of distinctive search techniques, and keywords will be customized correspondingly. Hand searching will be used besides electronic searches and will involve searching through the reference list. Based on eligibility criteria, domains will be set. These domains will be joined with the Boolean operators “AND” and “OR”. The filters will be applied as dates between 2020/01/01 and 2023/12/31 (the last three years); longitudinal or cohort studies (also short-term cohort studies); and cross-sectional studies; humans; printed in English only. The search strategy will be developed by two independent investigators (NJ and RC), and an expert reviewer (KS) will peer-examine the comprehensiveness of the search approach. A six-member team, all with clinical, scientific, and public health expertise in chronic COVID conditions and evidence synthesis, contributes to the study's selection for the review.

At level one, articles that fit the search terms will be selected based on abstract and title assessment by two independent investigators (NJ and RC). At level second, the remaining articles will be screened by abstract by three researchers separately (NJ, RC, and AG). At level third, the remaining articles will be selected by full text regarding eligibility criteria and study objective relevance by three researchers (NJ, RC, and AG). At level four, three researchers (KS, DS and DM) will evaluate all articles to ensure their relevance to the review objective and eligibility criteria. AG, KS, and DS will cross-check the eligibility of articles. Missing data will be addressed by first assessing the extent and details of missing information. The investigators will attempt to contact the study authors for the missing data. Studies may be excluded or analyzed based on available information if data cannot be retrieved. Methods like imputing missing values, using statistical models (e.g., random-effects meta-analysis), and conducting sensitivity analyses will be employed. We will document missing data, and any assumptions made in the review process. Risk of bias assessment will be performed and effect of missing data on the review's findings will be discussed transparently in the results.

All disagreements will be discussed with the entire review team. When discrepancies arise, they will be resolved through discussion and consensus. If necessary, a third reviewer or a senior investigator (KS) can mediate the decision. The authors of the included studies will be contacted if eligibility criteria remain elusive after a review by the entire review team. The final inclusion of the study, accompanied by reasons for exclusion, will be presented in a PRISMA (Preferred Reporting Items for Systematic Review and Meta-analysis) flow chart. The Microsoft Excel and EndNote 21 reference software will be used to record the data. Search results are deduplicated manually and with the assistance of EndNote 21 software.

### 2.3 Data collection process and data items

The following data will be included: data associated with the article; sociodemographic data of the population; study design and setting; clinical history of COVID-19 infection and vaccination; associated comorbidities and various symptoms of long COVID. A coded data extraction sheet will be developed to transform designated data into categorical data. The data extraction sheet will be pilot tested in the first five studies. Two reviewers (NJ and RC) will extract data separately from all the included articles and cross-compare them at review completion. In the event of discrepancies, an expert (KS) will be consulted.

### 2.4 Bias assessment in individual studies

To reduce the risk of selection bias, investigators will follow established standards for choosing studies, using a multi-step review process with three independent reviewers. Any differences in decisions will be resolved through discussion. The systematic review will document the study selection process with a PRISMA (Preferred Reporting Items for Systematic Reviews and Meta-Analyses) flow diagram to convey transparency in the selection process. The included studies will be assessed for quality using suitable tools, and a sensitivity analysis will be conducted at the end. Three more experienced independent investigators will perform the quality assessment. For cross-sectional and longitudinal or cohort studies, the quality assessment tool for observational cohort and cross-sectional studies (NIH) will be used to assess the risk of bias at the study level (11). The narrative synthesis will include an overview of the bias risk. The evidence will be summarized using the Quality Assessment Tool for Observational Cohort and Cross-Sectional Studies (NIH Tool). Considering the NIH tool evaluation, the data characteristics will be regarded as good, average, and poor. The entire review group will contribute to the quality of the evidence. There will be a summarized presentation of the findings (12).

### 2.5 Bias in studies

Publication bias will be estimated using Begg's test and Egger's test.

### 2.6 Data synthesis and meta-analysis estimation

All included articles will be studied in a narrative synthesis. Outcome data will be comprehensively collected from each study and presented in tabular form. The results section will document significant clinical and nonclinical confounders as descriptive summaries. A meta-analysis will be conducted contingent upon the availability and suitability of data. If sufficient and comparable quantitative data are identified across the included studies, statistical synthesis will be undertaken. Subgroup analyses and sensitivity analyses will be performed to manage confounders, alongside details of study characteristics, inclusion/exclusion criteria, and statistical adjustments will be reported. Potential residual confounders, those not fully accounted for, will be acknowledged as limitations of the evidence. MedCalc software (version 23.0.8) will assist a meta-analysis to assess the pooled prevalence of long COVID condition and will map the symptoms using quantitative data provided by individual studies. The upper limit of statistical significance will be set at *p* = 0.05. Heterogeneity between studies will be estimated and reported using the *I*^2^ index. Publication bias will be evaluated using statistical tests to ensure the integrity of the findings. In the absence of adequate data, a narrative synthesis will be performed to summarize the findings systematically and transparently.

### 2.7 Expected outcome

The primary aim of this research is to determine the prevalence of long COVID conditions in India. This requires a thorough examination of the symptoms and problems associated with long COVID, with a special emphasis on the severity and frequency of these outcomes. Furthermore, the study intends to explore broader aspects by investigating the link between long COVID and demographic factors like age and gender. In addition, the study will examine potential correlations with related comorbidities to acquire a more detailed understanding of the varied impact of prolonged COVID on different demographic groups and health conditions in India.

## 3 Discussion

Long COVID, also known as post-acute sequelae of SARS-CoV-2 infection, is a complex and multifaceted condition affecting individuals long after their initial COVID-19 disease (13). The condition's unpredictability and the spectrum of symptoms pose significant challenges in diagnosis, treatment, and management. However, the situation and understanding of long COVID are continuously evolving, and it's critical to stay current with the most recent information from dependable sources. The mapping of long COVID in India involves several crucial aspects. To start with, understanding the prevalence and distribution of long COVID symptoms at different ages and genders is fundamental. In the present systematic review and meta-analysis, we intend to study the long-term effects of COVID-19, with a special focus on the burden of long COVID among the Indian population. Our methodology ensures a comprehensive and unbiased selection of studies, providing valuable insights into this emerging health issue. This review will emphasize providing updated information on the prevalence of long COVID conditions in India and identifying factors such as age, gender, preexisting conditions, and vaccination status that could play a role in understanding susceptibility and severity. Furthermore, this meta-analysis and systematic review will list all the lingering effects of COVID-19 infection. This review will use a detailed methodology and examine a wide range of variables, considering several secondary outcomes and mapping long COVID prevalence as a primary outcome.

In Summary, this systematic review and meta-analysis will provide a thorough understanding of the long-term effects of COVID-19 on the Indian population. By employing rigorous methodologies and addressing potential biases, we will discover valuable insights that can inform healthcare interventions and future research in this critical area.

## 4 Strength and limitation

The existing meta-analysis and systematic review has several key strengths: (1) it attempts to conduct a meta-analysis (contingent upon the availability and suitability of data); (2) it incorporates empirical data (longitudinal or cohorts), case-control studies, and cross-sectional studies; (3) it searches a wide range of journals and databases; and (4) it investigates factors affecting long COVID severity (5) it will offer relevant data to support policy decisions. Limiting the inclusion criteria to English papers may result in language bias, which needs to be considered a limitation. In addition, creating a standardized framework for diagnosing and categorizing long symptoms of COVID can aid in accurate mapping.
